# Influence of Audiovisual Training on Horizontal Sound Localization and Its Related ERP Response

**DOI:** 10.3389/fnhum.2018.00423

**Published:** 2018-10-23

**Authors:** Yuexin Cai, Guisheng Chen, Xiaoli Zhong, Guangzheng Yu, Hanjie Mo, Jiajia Jiang, Xiaoting Chen, Fei Zhao, Yiqing Zheng

**Affiliations:** ^1^Department of Otolaryngology, Sun Yat-sen Memorial Hospital, Sun Yat-sen University, Guangzhou, China; ^2^Institute of Hearing and Speech-Language Science, Sun Yat-sen University, Guangzhou, China; ^3^Acoustic Laboratory, Physics Department, South China University of Technology, Guangzhou, China; ^4^Department of Speech Language Therapy and Hearing Science, Cardiff Metropolitan University, Cardiff, United Kingdom; ^5^Department of Hearing and Speech Science, Xinhua College, Sun Yat-sen University, Guangzhou, China

**Keywords:** audiovisual training, event related potential (ERP), late component, P400, N500

## Abstract

The objective was to investigate the influence of audiovisual training on horizontal sound localization and the underlying neurological mechanisms using a combination of psychoacoustic and electrophysiological (i.e., event-related potential, ERP) measurements on sound localization. Audiovisual stimuli were used in the training group, whilst the control group was trained using auditory stimuli only. Training sessions were undertaken once per day for three consecutive days. Sound localization accuracy was evaluated daily after training, using psychoacoustic tests. ERP responses were measured on the first and last day of tasks. Sound localization was significantly improved in the audiovisual training group when compared to the control group. Moreover, a significantly greater reduction in front-back confusion ratio for both trained and untrained angles was found between pre- and post-test in the audiovisual training group. ERP measurement showed a decrease in N1 amplitude and an increase in P2 amplitude in both groups. However, changes in late components were only found in the audiovisual training group, with an increase in P400 amplitude and decrease in N500 amplitude. These results suggest that the interactive effect of audiovisual localization training is likely to be mediated at a relatively late cognitive processing stage.

## Introduction

The ability to correctly localize sounds is an important feature of the auditory system and is directly associated with the ability to extract binaural information from surrounding sounds (Ahveninen et al., 2014). To accurately localize a sound, many acoustic cues may be needed. These include monaural cues such as spectral cues (determined by the interaction of the sound with the body, torso, head and shape of the external pinna), and binaural cues such as interaural-time difference (ITD) and interaural-level difference (ILD; which result from time and intensity differences between sounds arriving at the two ears; Bregman, 1990; Blauert, 1997). These cues are integrated in the brain and help to form and adjust individualized spatial map and related cortex and sub-cortex structures (Grothe et al., 2010; Ahveninen et al., 2014).

A number of studies have indicated that the ability to detect fine differences in binaural information is affected by hearing loss resulting in a deficit in the perception and analysis of the frequency and temporal information in sound inputs (Grieco-Calub and Litovsky, 2012; Rothpletz et al., 2012; Glyde et al., 2013; Cai et al., 2015). The study by Cai et al. (2015) found that sound discrimination ability was decreased in subjects with hearing loss and was associated with cortical compensatory changes. As a result, change to central reorganization and plasticity associated with hearing impairment may be one of the reasons for poor sound localization (Keating and King, 2013; Kral et al., 2016).

Studies on humans and animals with normal hearing show widespread multisensory influences on auditory cortical processing (Bizley and King, 2012; Isaiah et al., 2014). In particular, spatial selectivity in the auditory cortex is enhanced by the presence of spatially coincident visual stimuli(Bizley and King, 2008). This suggests that audiovisual training might be a vital tool for improving the capability of sound localization (Zahorik et al., 2006; Strelnikov et al., 2011; Majdak et al., 2013; Kuk et al., 2014). To reinforce this, the study by Strelnikov et al. (2011) found that 5 days of audiovisual training significantly improved sound localization performance when compared to only auditory training in healthy participants in monaural conditions. However, there is an absence of literature on the effect of audiovisual training in virtual acoustic condition, even though the techniques are widely used and have proved to be a more cost efficient approach for psychoacoustic sound localization testing (Strelnikov et al., 2011).

Furthermore, it remains unclear what neural mechanisms are behind the sound localization behavioral enhancement gained through training, this in turn is preventing researchers from improving the training paradigm. It is generally accepted that the perceptual skills needed for sound localization improve and produce behavioral enhancement when individuals repeatedly perceive the sound source during training. Training supports the subjects’ perceptual learning (Majdak et al., 2013) and related cortical plasticity changes of the neural circuit (Bruns et al., 2011; Powers et al., 2012; Harré, 2013). Among all the different kinds of training it seems that multisensory training in particular, with its activation of several cortical areas and multisensory interaction effect on learning, has the best effect on improving localization performance and shortening the necessary training duration (Shams and Seitz, 2008).

However, the neural correlates of audiovisual training remain controversial. Some studies report that training effect is mediated in early stage sensory processing. Bruns et al. (2011) demonstrated that an auditory evoked event-related potential (ERP) at 100 ms post-stimuli was modulated by disparity audiovisual training (the behavioral ventriloquism after effect). In contrast, other studies suggest that improvement in detection of sensory stimuli is mediated in late stage cognitive processing. Li et al. (2010) found that enhancement of auditory detection by audiovisual training was associated with ERP responses at 280–300 ms and 300–320 ms.

The present study aimed to combine behavioral and neurophysiological tests to estimate the efficiency of an audiovisual training program and its underlying neural mechanisms. It was hypothesized that this multisensory training protocol would improve sound localization performance significantly when compared to a uni-sensory auditory training method. Mean absolute error (MAE) and front-back confusion ratio were calculated to determine each subject’s sound localization accuracy and the spatial map distortion severity (Zahorik et al., 2006). To uncover the neural mechanisms, Electroencephalography (EEG) and extracted ERP components were recorded to identify the processing stage of the effect of audiovisual training on sound localization (Yang et al., 2007; Polezzi et al., 2008; Joos et al., 2014; Bakker et al., 2016).

## Materials and Methods

### Participants

Fourteen right-handed healthy volunteers participated in the study, eight men and six women, ranging in age from 22 to 25 years (Mean age = 23.07 years; SD = 1.072 years). No participants had taken part in any similar study previously. They were assigned to the training or control group randomly before the study. This study was carried out in accordance with the recommendations of the Institution Review Board of The Sun Yat-sen Memorial Hospital at Sun Yat-sen University of China. The protocol was approved by the Institution Review Board of The Sun Yat-sen Memorial Hospital. All subjects gave written informed consent in accordance with the Declaration of Helsinki. All participants had hearing thresholds measured using a pure tone audiometer (PTA) before the experiment on day 1. The audiological results showed average hearing thresholds, ranging from 250 Hz to 8,000 Hz at better than 20 dB HL.

### Apparatus

A Lenovo-based personal computer major controller unit (MCU), with a MAYA22USB sound card (ESI Audiotechnik GmbH, Germany) via a pair of high quality Sennheiser IE80 earphones (Sennheiser, Germany) was used to present the spatial auditory stimuli to the listeners. The visual stimuli signal unit consisted of an AT89S52C microcontroller and a series of red LEDs. The LEDs were placed at different azimuth angles and marked with direction of sound signals and would light up during audiovisual training. All the experiments were conducted in a sound attenuated, electrically shielded room (324 cm × 234 cm × 197 cm).

### Stimuli

The auditory stimulus for all portions of the experiment was white noise with a frequency ranging from 0.02 kHz to 20 kHz. Signal duration was 300 ms, including 10 ms rising and falling time. Interstimulus interval was chosen randomly from 3 s to 4 s at approximately 60 dB SPL.

All stimuli delivered bilaterally from different positions in the horizontal plane were convolved by HRTF data, with azimuthal sampling of 5°, was measured from a Knowles Electronic Mannequin for Acoustic Research (KEMAR) under a listening room in South China University of Technology (Xie, 2013), in which the signal-noise-ratio is about 30 dBA and reverberation time is about 0.15 s. Since the measurement was not held in an anechoic chamber, the room reflections have been eliminated by using a time window (Zhong and Xie, 2009; Xie, 2012; Cai et al., 2015).

### Study Design and Procedure

#### The Procedure of Pre-phase Study

Full instructions were given before the localization test in order to avoid confusion when perceived localizational changes via headphones during the experiment. In addition, all participants were given localization task training using various stimuli from different directions in order to familiarize themselves with the localization test procedure and adapt to the HRTF-convolved sounds, which improved the reliability and accuracy of the tests.

**Figure 1:**
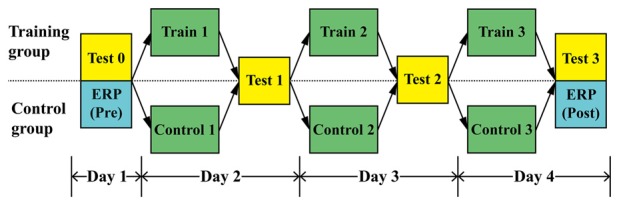
Phases in the experimental design for the training and control groups.

**Figure 2:**
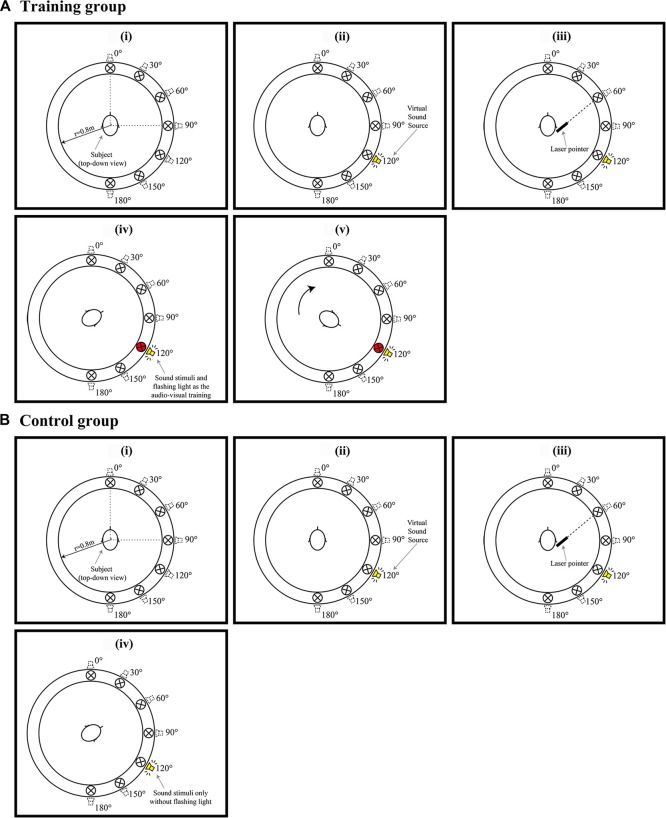
**(Ai–v)** Example of a trail when the audiovisual training conducted. **(Bi–iv)** Example of a trail when Only auditory training conducted. Red circles in **(Aiv,v)** represent a visual LED indicator of the correct sound position paired with a spatialized auditory stimulus. **(Av)** shows the participants were subsequently requested to response and look the visual LED indicator at that direction and listen the auditory stimuli with LED presented simultaneously and repeatedly 10 times.

Figure 1 shows that the entire study took 4 days to complete. During Day 1 (Pre-phase), all participants were required to complete a sound localization test (Test 0) and an ERP test. For sound localization (Test 0), participants would hear signals from 13 directions (0, 15, 30, 45, 60, 75, 90, 105, 120, 135, 150, 165, 180°) and they were required to perceive the direction and indicate it with a laser pointer. Considering the facts of so-called “right ear advantage” and dominance for temporal processing of left hemisphere, together with people’s preference when perceiving the auditory stimuli (Hall et al., 2002; Richter et al., 2013; Payne et al., 2017; Tai and Husain, 2018), only right-side sounds were used in the experiment, as shown in Figures 2A,B. In the localization test, to verify the participants was able to localize accurately, participants were asked to indicate the directions until they were confident that they had point to the direction as accurately as possible.

Considering of participant’s compliance and attention span, signals from various directions were emitted one at a time in a pseudorandom sequence, with each direction emitting four times, and no sounds successively emitting from the same direction. There is a total of 13 angles, and thus approximately 40–45 min are needed. For the ERP test, participants sat comfortably in a quiet dark room with arms and wrists relaxed. To avoid any motor activity effects on EPR recording, they were required to localize the sound only by moving a finger to point out the direction. The purpose of this requirement is to avoid any motor activity effects on EPR recording and keep participants’ attention to the stimuli. Therefore, the entire procedure of Pre-phase study took more than one and a half hour. There were several breaks between the tests.

#### Procedure for Visual-Auditory Training

Participants randomly allocated to the training group completed the audiovisual training program in the three consecutive days after Pre-phase study (Tests 1, 2 and 3, respectively). Figure 2 shows an example of the steps for the training program, they are:

1. The participants were initially tested to locate the sound emitting from seven individual directions (0°, 30°, 60°, 90°, 120°, 150°, 180°). Sounds from different directions were emitted in a pseudorandom sequence, with each direction appearing four times (Figures 2Ai–iii).
2. Participants were then informed the correct direction of the target signals by lighting up the LEDs (Figure 2Aiv).
3. The participants were subsequently requested to look in the direction of the LED and listen to the auditory stimuli when both HRTF signal and the LED were presented simultaneously and repeatedly 10 times (Figure 2Av).
4. The participants were tested with the next target signal soon after the audiovisual training.

The procedure for the training program was repeated during Tests 1, 2 and 3.

#### Procedure for Control Group

In contrast, participants in the control group also completed a series of auditory training in the three consecutive days after Pre-phase study (Tests 1, 2 and 3, respectively; Figures 2Bi–iii), they did not receive either feedback of the correct direction or visual reinforcement. As shown in Figure 2Biv; only auditory stimuli were presented repeatedly for 10 times.

After completing Test 3 on day 4, all participants undertook an ERP test as they did on day 1. KEMAR HRTF signals emitting from 0, 45, 90, 135, 180° were used during the ERP measurement.

### ERP Recording

EEG data was recorded using a 128-channel Geodesic Sensor Net and was amplified electronically using a Net Amps 300 instrument (Electrical Geodesics Inc., Eugene, OR, USA). Before each ERP test, all electrode impedances were measured and kept below 50 kΩ. The effective sampling rate was 500 Hz and electrodes with reference to the average signal of all electrodes. Eye movements were recorded using electrodes positioned above and below the right eye and near the left and right outer canthi. The whole task consisted of a total of 750 auditory stimuli with an inter-stimulus interval (ISI) varying from 750–900 ms. To minimize possible artifacts caused by motor responses, in the present study, the participants were instructed and requested to minimize blinking and any bodily movements, subjects were required to keep attention to the auditory stimuli.

### Data Processing

#### Behavioral Data

After the sound localization test, MAE and front-back confusion ratio were calculated according to target azimuth and perceived azimuth from all phases of the experiment for further analysis. MAE was defined as the mean absolute deviation of the response angle from the target angle, in degrees, indicating how accurately a subject localized a sound. Front-back confusion ratio represented the ability to discriminate sounds from the front and back. It was calculated by the total number of front-back reversal responses divided by the number of total responses (Zahorik et al., 2006; Zhong et al., 2015).

#### ERP Data

EEG data were analyzed offline using Net Station software (Electrical Geodesics Inc., Eugene, OR, USA). The signals were digitally bandpass-filtered from 0.05 to 20 Hz. The continuous EEG was then parsed into 1,000 ms segments with a 100 ms pre-stimuli baseline. Trials affected by eye-movements of more than 55 μV, eye blinks of more than 140 μV, or bad channels artifacts of more than 200 μV amplitude were rejected. After that, data were averaged, re-referenced to average signal of all electrodes, and baseline-corrected across all target sound locations. Because auditory response and audiovisual integration effect was found mainly at centromedial distribution (Talsma and Woldorff, 2005), FCz was selected for further statistical analysis.

### Statistical Analysis

Values of localization accuracy and ERP response across conditions were analyzed using a repeated measures analysis of variance (RM-ANOVA) in SPSS (v.16.0). The MAE of localization were subjected to a three-way RM-ANOVA, including factors of group (audiovisual training group, only auditory control group), time (test 0, 1, 2, 3) and angle (trained angle, untrained angle). Regarding the ERP responses, mean amplitudes of the time windows for the ERP components (N1: 50–150 ms; P2: 150–250 ms; P400: 300–400 ms; N500: 400–500 ms) were used in statistical analysis. Three-way RM-ANOVAs was performed including factors of group (audiovisual training group, only auditory control group), time (pre- and post-test) and angle (trained angle, untrained angle) for N1, P2, P400 and N500 amplitude. Probability values were corrected using Greenhouse-Geisser when the assumption of sphericity was violated. Further *Post hoc* comparison were Bonferroni corrected. In addition, Chi-square statistics was used to compare the front-back confusion ratio between conditions, including factors of time, angle and group. All the chi-square statistics were done with Bonferroni correction.

## Results

### Demographic Data

There was no significant difference in age (*t* = −1.795, *p* = 0.098) or gender (Fisher’s Exact Test, *p* = 1.000) between the two groups (audiovisual training and only auditory control).

### Behavioral Data

#### Mean Absolute Error (MAE)

A set of test-retest reliability analyses for MAE of total, trained and untrained angles on different time for both groups were conducted. The results for control and training groups were showed in Tables 1, 2, respectively. These analyses revealed that all the intra-class correlation was larger than 0.5 while all the *P*-values for the analyses were smaller than 0.05. Therefore, all the results indicate that the data is repeatable and reliable.

**Table 1:** Result of test-retest reliability analyses for control group.

Items	Intra-class correlation	95% Confidence interval		*P* value
		Lower bound	Upper bound	
Total angles:				
Trial 1 vs. Trial 4
Test 0	0.860	0.604	0.955	0.000
Test 1	0.794	0.453	0.932	0.000
Test 2	0.767	0.397	0.923	0.001
Test 3	0.831	0.535	0.945	0.000
Trained angles:				
Trial 1 vs. Trial 4				
Test 0	0.848	0.353	0.972	0.004
Test 1	0.852	0.365	0.973	0.004
Test 2	0.880	0.459	0.978	0.002
Test 3	0.827	0.288	0.968	0.006
Untrained angles:				
Trial 1 vs. Trial 4				
Test 0	0.748	− 0.015	0.960	0.027
Test 1	0.852	0.273	0.978	0.007
Test 2	0.728	− 0.059	0.957	0.032
Test 3	0.886	0.397	0.983	0.004

*Note: Trial 1 means the first trial in each condition. Trial 4 means the last trial in each condition*.

**Table 2:** Result of test-retest reliability analyses for training group.

Items	Intra-class correlation	95% Confidence interval		*P* value
		Lower bound	Upper bound	
Total angles:				
Trial 1 vs. Trial 4				
Test 0	0.914	0.742	0.973	0.000
Test 1	0.801	0.469	0.935	0.000
Test 2	0.534	0.002	0.830	0.025
Test 3	0.595	0.092	0.856	0.012
Trained angles:				
Trial 1 vs. Trial 4				
Test 0	0.845	0.343	0.971	0.004
Test 1	0.838	0.320	0.970	0.005
Test 2	0.918	0.600	0.985	0.001
Test 3	0.941	0.698	0.990	0.000
Untrained angles:				
Trial 1 vs. Trial 4				
Test 0	0.776	0.053	0.965	0.020
Test 1	0.864	0.314	0.980	0.006
Test 2	0.887	0.402	0.983	0.004
Test 3	0.672	−0.167	0.947	0.049

*Note: Trial 1 means the first Trial in each condition. Trial 4 means the last trial in each condition*.

##### Total Angles

**Figure 3:**
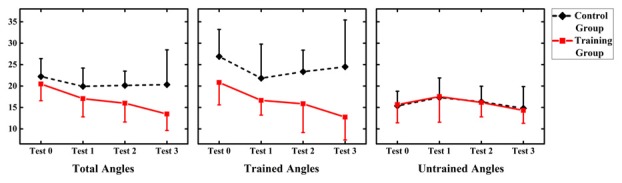
Summary of mean absolute error (MAE) for the audiovisual training and control groups for three types of angles (total angles, trained angles and untrained angles). Error bars show the magnitude of one standard error.

Values of MAE performance were subjected to a 2 (group: audiovisual training, only auditory control) × 4 (time: test 0, 1, 2, 3) RM-ANOVA. The results demonstrated a main effect of factor “Group” (*F*_(1,12)_ = 5.366, *p* = 0.039), which revealed that MAE for only auditory control group (mean = 20.302, SD = 7.166) was significantly larger than that for audiovisual training group (mean = 16.137, SD = 7.166; Figure 3).

However, no significant main effect of factor “Time” or interaction between “Time” and “Group” was shown (*F*_(3,36)_ = 2.825, *p* = 0.052; *F*_(3,36)_ = 0.998, *p* = 0.405, respectively).

##### Types of Angles: Trained vs. Untrained

###### ANOVA

In order to investigate the generalized effect of training on untrained location, values of MAE performance were subjected to a 2 (group: audiovisual training, only auditory control) × 4 (time: test 0, 1, 2, 3) × 2 (angle: trained angle, untrained angle) RM-ANOVA. There was significant main effect of angles (*F*_(1,12)_ = 9.895, *p* = 0.008), with MAE for trained angles (mean = 19.804, SD = 7.593) larger than that for trained angles (mean = 16.040, SD = 4.112). However, no significant effect of time and group was found (*F*_(3,36)_ = 2.583, *p* = 0.068; *F*_(1,12)_ = 4.671, *p* = 0.052, respectively). Significant interactions were found between angles and group (*F*_(1,12)_ = 6.728, *p* = 0.023) and between angles and time (*F*_(3,36)_ = 3.604, *p* = 0.025). Interaction was further broken down by angle.

###### Trained Angles

Values of MAE performance at trained angles were subjected to a 2 (group: audiovisual training, only auditory control) × 4 (time: test 0, 1, 2, 3) RM-ANOVA. Significant main effect of group (*F*_(1,12)_ = 7.313, *p* = 0.019) demonstrated a larger MAE for only auditory control group (mean = 24.194, SD = 7.560) than that for audiovisual training group (mean = 16.511, SD = 5.812). There was significant effect of time (*F*_(3,36)_ = 2.583, *p* = 0.48) showed that MAE was significantly reduced in post-test (Day 4 = 23.36 ± 1.53) than MAE in pre-test (17.79 ± 2.18) at trained angles. Besides, interaction between time and group were found significant (*F*_(3,36)_ = 2.941, *p* = 0.046). Further analysis was broken down by factor of time and the independent-sample *t*-test revealed no significant difference between the two groups in test 0 and 1 (*t* = −1.986, *p* = 0.070; *t* = −1.744, *p* = 0.107, respectively), while a significant difference was suggested between the two groups in test 2 and 3 (*t* = −2.268, *p* = 0.043; *t* = −2.670, *p* = 0.020, respectively), revealing a larger MAE for only auditory control group (mean = 23.357, SD = 5.023; mean = 24.5, SD = 10.936, respectively) than that for audiovisual training group (mean = 15.920, SD = 6.720; mean = 12.754, SD = 5.324, respectively) in test 2 and 3.

###### Untrained Angles

Values of MAE performance at untrained angles were subjected to a 2 (group: audiovisual training, only auditory control) × 3 (time: test 0, 1, 2, 3) RM-ANOVA.

No significant main effect of group and time (*F*_(1,12)_ = 0.138, *p* = 0.717; *F*_(3,36)_ = 0.303, *p* = 0.823, respectively), as well as no interaction between time and group were found (*F*_(3,36)_ = 0.052, *p* = 0.984).

#### Front-Back Confusion Ratio

**Figure 4:**
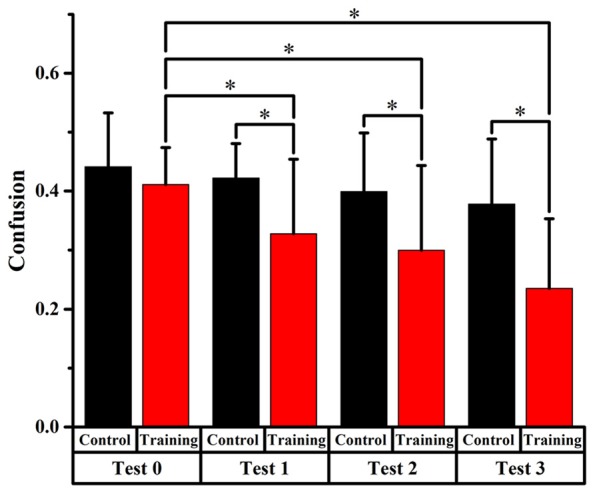
Comparison of front-back confusion ratio for the audiovisual training and control groups between pre- and post-test at total angles. *Meant the difference was significant (*P* < 0.05).

**Figure 5:**
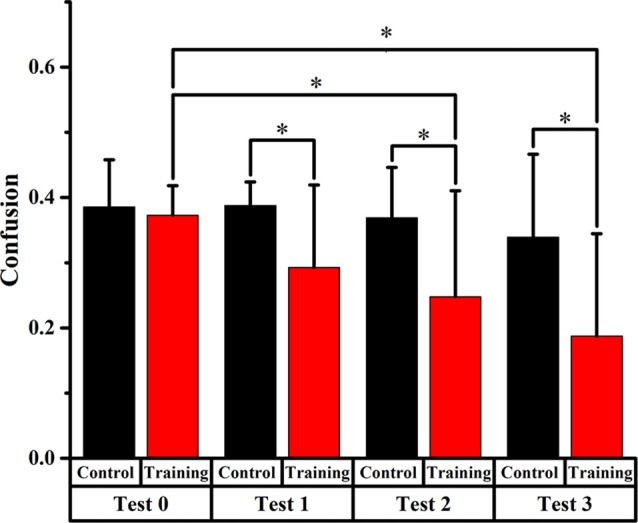
Comparison of front-back confusion ratio for the audiovisual training and control groups between pre- and post-test at trained angles. *meant the difference was significant (*P* < 0.05).

**Figure 6:**
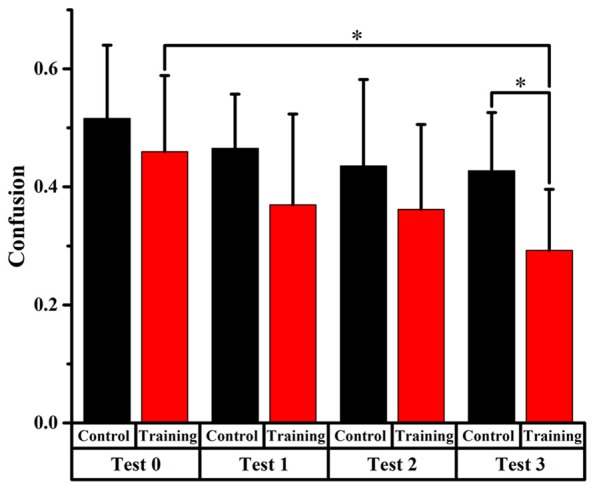
Comparison of front-back confusion ratio for the audiovisual training and control groups between pre- and post-test at untrained angles. *meant the difference was significant (*P* < 0.05).

Figures 4, 5, 6 display the proportion of front-back reversal responses for all listeners from the two groups for test 0 vs. test 1, test 0 vs. test 2, and test 0 vs. test 3 and the analysis of differences in front-back confusion ratio between the two groups in every phase for total angles, trained angles and untrained angles, respectively.

##### Total Angles

Front-back confusion ratio for the two groups decreased as time passed by. For the training group, the statistical analysis showed significant differences (*p* < 0.0125, Bonferroni correction) between test 0 and test 2, 3, respectively (*x*^2^ = 10.859, *p* = 0.001; *x*^2^ = 28.519, *p* < 0.001, respectively), which revealed that the front-back confusion ratio decreased as the experiment conducted (0.411, 0.300, 0.235 for test 0, 2, 3, respectively). For the control group, however, no significant difference between test 0 and test 1, 2, 3 was found (see Figure 4).

##### Trained Angles

For the training group, the statistics analysis showed significant differences (*p* < 0.0125, Bonferroni correction) of front-back confusion between test 0 and test 2, 3, respectively (*x*^2^ = 8.070, *p* = 0.005; *x*^2^ = 18.919, *p* < 0.001, respectively), which revealed that the front-back confusion ratio decreased as the experiment conducted (0.373, 0.248, 0.188 for test 0, 2, 3, respectively). However, no significant difference was shown between test 0 and test 1 for the training group and between test 0 and test 1, 2, 3 for the control group (see Figure 5).

Furthermore, the Chi-square statistics showed that except for test 0(*x^2^* = 0.066, *p* = 0.797), the difference in front-back confusion ratio between the two groups was significant for test 1, 2 and 3 (*x*^2^ = 3.851, *p* = 0.050; *x*^2^ = 6.700, *p* = 0.010; *x*^2^ = 11.717, *p* = 0.001, respectively; see Figure 5).

##### Untrained Angles

Only significant difference (*p* < 0.0125, Bonferroni correction) of front-back confusion was found between 0 and test 3 in the audiovisual training group (*x*^2^ = 10.805, *p* = 0.001), which revealed that the front-back confusion response was reduced in post-test than that in pre-test (0.293 and 0.460 for test 3 and 0, respectively). No significant difference was found in the auditory control group (see Figure 6). The Chi-square statistics showed that only significant difference found on test 3 in comparison between the two groups (*x*^2^ = 6.195, *p* = 0.013), which revealed that the front-back confusion ratio for audiovisual training group (0.293 for test 3) was smaller than that for only auditory control group (0.427 for test 3) after 3-days’ training (see Figure 6).

### ERP Data

#### N1 Amplitude

For the N1 amplitude, RM-ANOVA revealed a significant effect from Time (*F*_(1,12)_ = 8.291, *p* = 0.014) with a stronger amplitude pre-test (mean = −0.982 μV, SE = 0.229 μV) than post-test (mean = −0.409 μV, SE = 0.296 μV). No significant effect was found for other factors or interactions between factors.

#### P2 Amplitude

**Figure 7:**
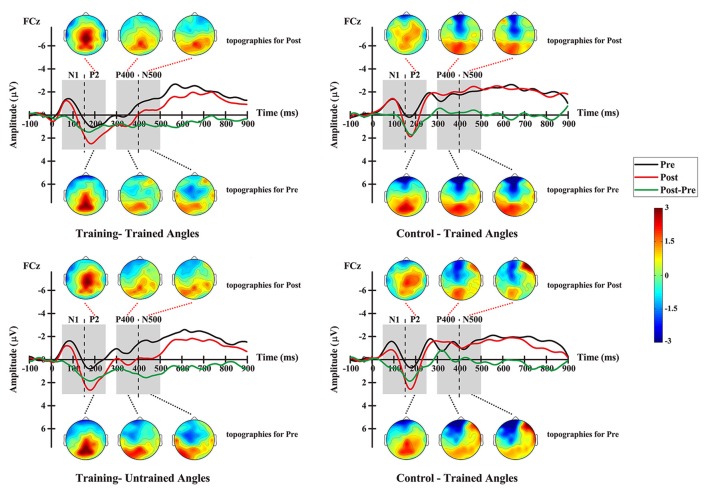
Grand-averaged ERPs elicited by auditory stimuli at electrode FCz and topographic maps at pre- and post-test for the training group (left column) and the control group (right column), and shown separately for trained angles (upper row) and untrained angles (lower row).

For the P2 amplitude, RM-ANOVA revealed a main significant effect of time (*F*_(1,12)_ = 17.690, *p* = 0.001), showing a stronger amplitude at the post-test (mean = 1.555 μV, SE = 0.346 μV) than at the pre-test (mean = 0.220 μV, SE = 0.371 μV; Figure 7). Additionally, the interaction between factor angles and factor group was found to be significant (*F*_(1,12)_ = 10.312, *p* = 0.007). No significant effect was found for other factors or interactions between factors.

For further analysis, interaction was broken down by factor of group. Therefore, values were subjected to 2 (angles: trained, untrained angles) × 2 (time: pre-, post-test) RM-ANOVA. The analysis for the training group revealed a statistically significant main effect of time (*F*_(1,14)_ = 6.402, *p* = 0.024), demonstrating a stronger amplitude at the post-test (mean = 2.088 μV, SE = 0.403 μV) than at the pre-test (mean = 0.646 μV, SE = 0.403 μV). The analysis for the control group showed a significant main effect of angles (*F*_(1,10)_ = 13.935, *p* = 0.004), revealing a stronger amplitude for untrained angles (mean = 0.715 μV, SE = 0.431 μV) than for trained angles (mean = 0.101 μV, SE = 0.478 μV).

#### P400 Amplitude

For the P400 amplitude, RM-ANOVA revealed a significant main effect of group (*F*_(1,12)_ = 10.950, *p* = 0.006), indicating a stronger amplitude for the training group (mean = −0.009 μV, SE = 0.296 μV) than for the control group (mean = −1.505 μV, SE = 0.342 μV; Figure 7). Moreover, the interaction between angles and group was found to be significant (*F*_(1,12)_ = 6.198, *p* = 0.028). No significant effect was found for other factors or interactions between factors.

Interaction was further broken down by factor of group. Values were subjected to 2 (angles: trained, untrained angles) × 2 (time: pre-, post-test) RM-ANOVA. For the training group, the analysis revealed a statistically significant main effect of time (*F*_(1,14)_ = 4.824, *p* = 0.045), demonstrating a stronger amplitude at the post-test (mean = 0.466 μV, SE = 0.305 μV) than at the pre-test (mean = −0.483 μV, SE = 0.305 μV). In addition, a significant effect of angle (*F*_(1,14)_ = 6.442, *p* = 0.024) was found for the training group, which means that trained angles elicited a greater P400 amplitude (mean = −0.283 μV, SE = 0.218 μV) than untrained angles (mean = −0.300 μV, SE = 0.269 μV). However, the analysis showed no significant effect for any factor or interaction between factors in the control group.

#### N500 Amplitude

For the N500 amplitude, RM-ANOVA revealed a main significant effect of the interaction between time and group (*F*_(1,12)_ = 5.300, *p* = 0.040). No significant effect was found for other factors or interactions between factors. For further analysis, repeated-measure ANOVAs were conducted for each group. The analysis for the training group revealed a statistically significant main effect of time (*F*_(1,14)_ = 14.291, *p* = 0.002), demonstrating a stronger amplitude at the pre-test (mean = −1.427 μV, SE = 0.188 μV) than at the post-test (mean = −0.267 μV, SE = 0.344 μV; Figure 7). However, the analysis showed no significant effect for any factor or interaction between factors in the control group.

## Discussion

The present study aimed to investigate the effect of audiovisual multisensory training on sound localization and the underlying neural mechanisms by conducting both behavioral and neurophysiological measurements. The results show that subjects from the training group improved their accuracy in sound localization significantly after 3 days’ training, but there was no significant improvement in the control group. In addition, the audiovisual training group showed a significantly greater decrease in front-back confusion ratio in comparison to the control group. Furthermore, the effect of reduction in front-back confusion was found to generalize to the untrained locations following audiovisual training. These results may be useful for practical application.

ERP measurement showed a decrease of the N1 amplitude and increase of the P2 amplitude in both audiovisual and only auditory training groups. However, a change of late components was only found in the audiovisual training group with an increase of the P400 amplitude and decrease of the N500 amplitude. These results suggest that 3 days of short-term training could also improve the sound localization and the interaction effect of audiovisual localization training is likely to be mediated at a relatively late cognitive processing stage.

Reductions in MAE and front-back confusion ratio were found for the multisensory training group with just 3 days short-term training. This indicates that improvement of localization accuracy and front-back discrimination can be achieved by audiovisual training. The improvement of localization accuracy was found for trained angles in the audiovisual training group, which is consistent with previous studies (Zahorik et al., 2006; Majdak et al., 2010). In addition, a significant reduction in front-back confusion was found after audiovisual training. The front-back confusion ratio was calculated by counting the number of front-back reversal responses and dividing it by the number of total responses. This result is in accordance with previous studies, indicating that listeners can effectively recalibrate to degraded spectral cues (i.e., caused by HRTF) as well as alignment of spatial representations to sound direction, particularly after audiovisual multisensory training (Zahorik et al., 2006; Kuk et al., 2014; Keating et al., 2016). An example of calibration changes in the perception of acoustic space is the ventriloquism aftereffect, i.e., individual sound localization shifts in accordance with this disparity after exposure to a consistent spatial disparity between auditory and visual stimuli (Recanzone, 1998; Lewald, 2002).

Furthermore, the findings of significant enhancements in front-back confusion measurement for both trained and untrained angles following audiovisual training are consistent with the findings by Zahorik et al. (2006). Their results showed that front-back confusion was significantly reduced at both trained and untrained locations with 2 days audiovisual training. Therefore, audiovisual training effect could spread to the untrained orientation and the enhancement of front-back confusion may be mediated by higher-level cognitive processing. This top-down effect suggests that audiovisual training may be used as an effective strategy for patients with unilateral hearing impairment, who have difficulty in locating the source in front or behind resulting from degraded binaural hearing cues. In addition, the improvement of front-back confusion for untrained angle was only found for audiovisual training. The possible reason may be attributed from the factors of spatial cues information and HRTF adaption (Zahorik et al., 2006). Because relatively simple analysis of sound level in the 3–7 kHz region can provide effective information as to whether the source is located in the front or back hemifield, we suggest that the improvements observed in front-back confusion following the audiovisual training may have resulted from improved processing of this spectral information (Zahorik et al., 2006). In addition, the localization error analysis required the processing of binaural spatial cues information (ITD and ILD). The distinction may explain why the improvement of recalibration to front-back cue can only occur in audiovisual training group for untrained angle.

Regarding the ERP study on sound localization, previous studies have found that changes of spatial cues (ITD, ILD, spectral cues) could evoke MMN response (100–130 ms) in auditory cortex (Altmann et al., 2017a,b; Frey et al., 2017). In the present study, neurophysiological results did not show significant changes in terms of the early ERP components of N1 either between pre- and post-tests or between audiovisual and auditory only training groups. Regarding the P2 component around 150–250 ms after stimuli, significant increase of amplitude was found for both groups in comparison between pre- and post-tests. These results may be an indication of perceptual learning, which is defined as experience-dependent enhancement of individual’s sensory behavioral ability (Hawkey et al., 2004). Previous studies have demonstrated that perceptual learning may modulate neural changes in early and late evoked potentials (Gold and Watanabe, 2010; Ahmadi et al., 2017). For example, Ahmadi et al. (2017) reported significant changes to C1, P1 and P3 components following visual perceptual training.

P400 is a late ERP component and is strongly modulated by attention, memory processing and decision making. Interestingly, in the present study, the amplitude of P400 was significant increased only in the audiovisual multisensory training group between pre- and post-tests. This result is consistent with finding by Li et al. (2010), who showed significant ERP responses were elicited at 280–300 ms and 300–320 after the presentation of stimuli. In addition, Talsma and Woldorff (2005) also showed that the audiovisual multisensory integration occurred on the ERP responses at multiple periods around 160, 250 and 300–500 ms after onset of stimuli. Therefore, auditory detection enhancement by audiovisual training may be mediated in the late processing stage rather than early stage sensory processes. Senkowski et al. (2007) also found multisensory integration effects beginning at 120–140 ms and together with a widespread network activation of occipital, temporal and frontal areas at longer latency (210–250 and 300–350 ms). It indicated that the multisensory integration for naturalistic objects was mediated at multiple early and late stages. However, the study by Kim et al. (2008) showed that the benefits of audiovisual training may result from audiovisual interaction at an early perceptual processing stage. One possibility would be effects of modality select attention that was only auditory stimuli and required to be attended in our study, whereas previous studies by Kim et al. (2008) and Fiebelkorn et al. (2011) required participants to attend both visual and auditory stimuli. It was known that audiovisual attention would facilitate and enhance the processing of stimuli. Talsma and Woldorff (2005) reported that when attention was directed to both modalities simultaneously, audiovisual interaction occurred in early sensory processing, whereas when only one modality was attended, the interaction processes was delayed to late processing stage.

Considering findings obtained from the spread of the reduction of front-back confusion by the effect of audiovisual training to untrained locations, the present study suggests that high cognitive level of cortical process (such as decision making) contribute to the sound localization enhancement of audiovisual training. However, effects of perceptual learning may not be ruled out due to significant changes of P2 between pre- and post-test in audiovisual training group. Therefore, the enhancement of sound localization results from audiovisual training may be possibly modulated at multiple late processing stages.

It is noteworthy that there are a few limitations in the present study. For example, due to the complexity and duration of the experiments as well as patient compliance issues, only a small sample size was recruited in the present study. Future longitudinal and large sample prospective research is needed to explore the long-term effect of audiovisual training, together with its associated central processing mechanism.

## Conclusion

The current study found that audiovisual localization training can significantly improve localization accuracy and reduced front-back confusion in virtual acoustic environment using virtual non-individualized HRTF. Significant improvement of sound localization accuracy was found for audiovisual training only at angle locations, while the significant reduction of front-back confusion by the audiovisual training effect can spread to untrained locations. Taken together with significant findings of ERP components of P2, P400 and N500, it suggests that the enhancement of sound localization by audiovisual training was modulated by higher level cognitive learning at multiple late processing stages.

## Data Availability

The original data that support the findings derived from this study can be requested by e-mailing yiqingzheng@hotmail.com.

## Author Contributions

YC and GC contributed conception and design of the study. HM, JJ and XC performed the experiments and collected the data. FZ designed the plan of analysis. XZ and GY performed the final analyses. YC and YZ drafted the manuscript and interpreted the results. All authors made substantive editorial contributions at all stages of manuscript preparation.

## Conflict of Interest Statement

The authors declare that the research was conducted in the absence of any commercial or financial relationships that could be construed as a potential conflict of interest.
